# Expulsion of rope-like material per rectum in an infant: a rare case report of pseudomembranous colitis

**DOI:** 10.1093/omcr/omag090

**Published:** 2026-06-08

**Authors:** Ahmad Hosiian, Mostafa Hassan, Ali ghanem, Mohamed Alafandi, Hayan Ghanem, Alaa Alani

**Affiliations:** Department of Infectious Diseases, Tartous National Children’s Hospital, Tartous, Syria; Department of Neurosurgery, Tartous University, Tartous, Syria; Department of Vascular Surgery, Latakia University, Latakia, Syria; Department of Radiology, Aleppo University, Aleppo, Syria; Department of Pathology, Tartous University, Tartous, Syria; Department of Dermatology, Tartous University, Tartous, Syria

**Keywords:** infectious diseases and tropical medicine, paediatrics

## Abstract

Pseudomembranous colitis (PMC) is a severe manifestation of *Clostridioides difficile* infection and is exceedingly rare in early infancy. We report a unique case of a 2-month-old male infant who presented with fever, profuse watery diarrhea, vomiting, and the passage of multiple rope-like colonic mucosal casts in the stool, initially raising concern for intestinal parasitosis. Laboratory investigations revealed marked leukocytosis, severe hyponatremia, and elevated inflammatory markers. Abdominal ultrasonography revealed diffuse thickening of the colonic wall. Histopathological examination of the expelled material confirmed a complete cast of necrotic colonic mucosa consistent with severe colitis. Despite an initial negative toxin assay, subsequent polymerase chain reaction (PCR) testing detected *C. difficile* toxin genes (tcdA and tcdB). The patient responded well to aggressive supportive care and intravenous antimicrobial therapy. This case highlights a rare and misleading presentation of PMC in early infancy and underscores the critical role of histopathology in achieving an accurate diagnosis.

## Introduction

*C. difficile* infection (CDI) is a well-recognized cause of gastrointestinal disease, most commonly presenting with diarrhea [1]. Pseudomembranous colitis (PMC) represents the severe end of the CDI spectrum and is characterized by the formation of adherent pseudomembranes on the colonic mucosa, usually in association with antibiotic exposure in adults [2]. In contrast, clinically significant PMC in infants—especially in very young patients without classic risk factors—is exceptionally rare, and its diagnosis is often challenging because of nonspecific symptoms such as fever and diarrhea [3]. The expulsion of rope-like colonic material has been described in non-scientific contexts; however, such findings are more plausibly explained as mucosal casts or inflammatory debris rather than parasitic organisms [4].

Here, we report a rare case of pseudomembranous colitis in a two-month-old infant presenting with expulsion of a complete colonic mucosal cast mimicking an intestinal parasite.

**Figure 1:**
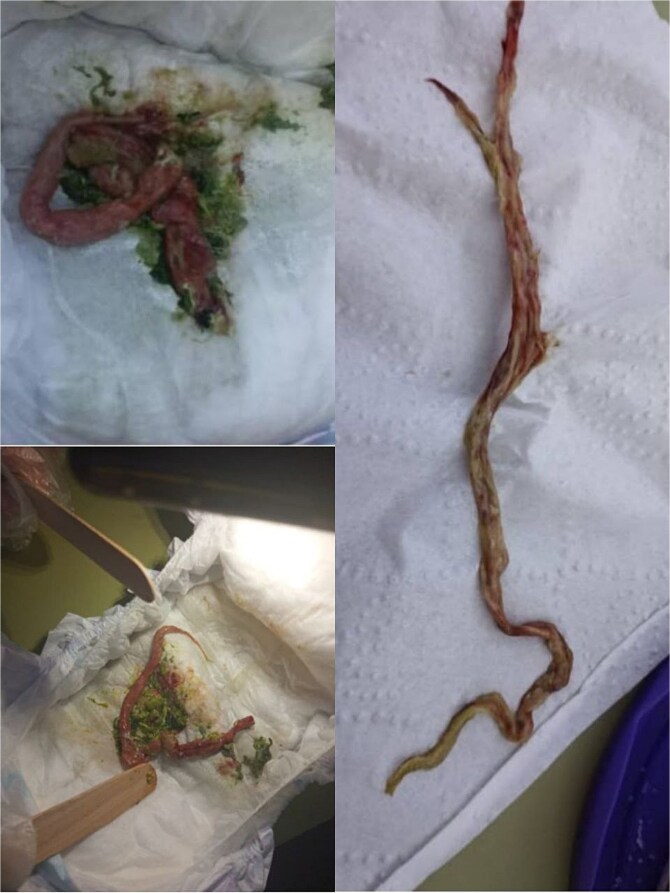
Macroscopic appearance of the rope-like material expelled per rectum.

**Figure 2:**
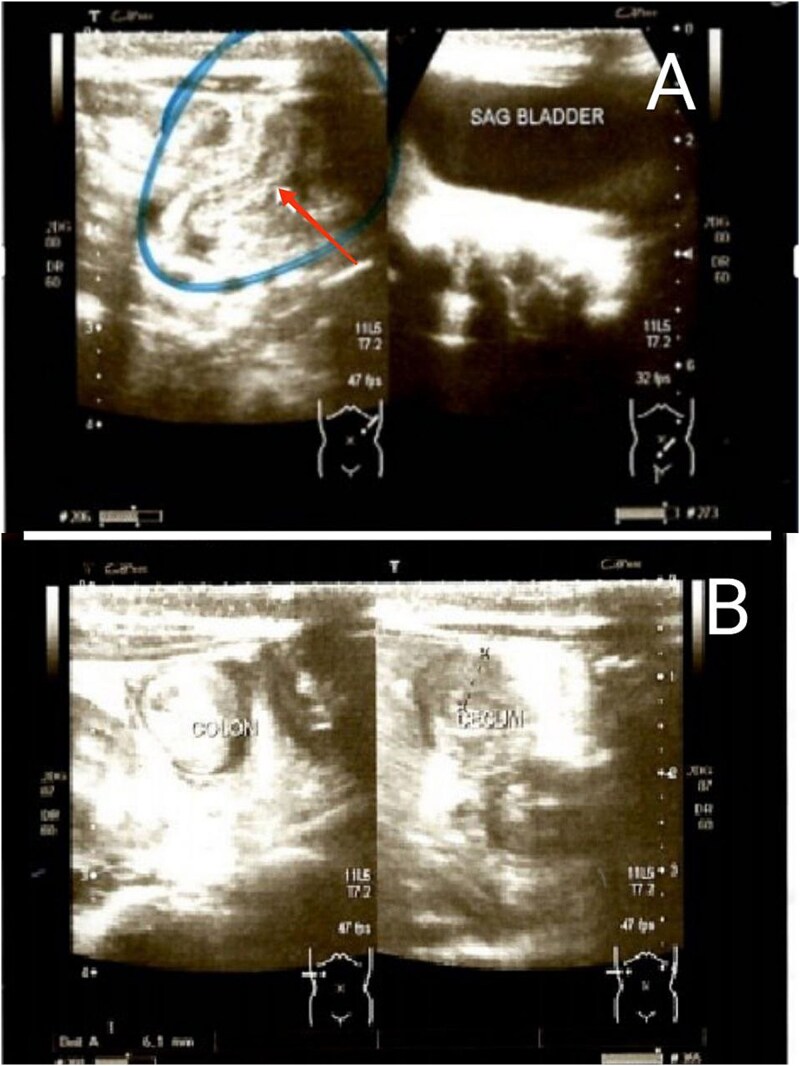
Ultrasonography findings (A) ultrasonography of the left hypochondriac and inguinal regions showing dilated small bowel loops (red arrow). (B) Ultrasonography of the right hypochondriac region demonstrating a high-positioned cecum with markedly thickened and edematous walls (approximately 6 mm).

**Figure 3:**
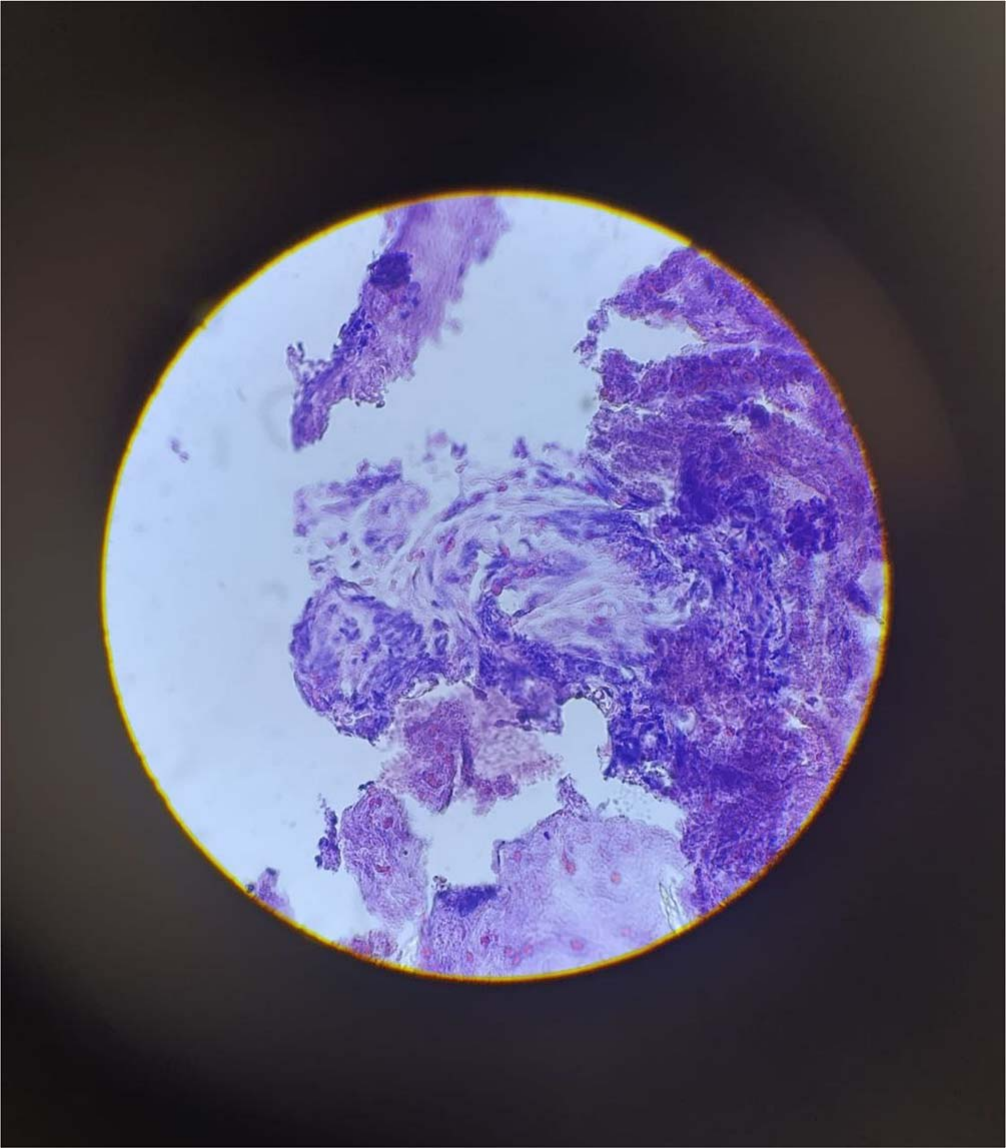
Histopathological image showing necrotic colonic mucosa and acute inflammatory infiltrates (H&E stain, 100x).

## Case report

A 2-month-old male infant from Syria presented with a 6-day history of fever, profuse watery diarrhea (>10 episodes per day), and recurrent vomiting. A notable feature was the passage of multiple rope-like structures, approximately 10 cm in length, in the stool for four consecutive days. (Figure 1) The infant was exclusively breastfed prior to presentation. The infant was born at term via uncomplicated vaginal delivery, with normal birth weight. There was no history of prematurity, NICU admission, or prior antibiotic exposure.

On admission, the infant appeared ill and lethargic. Vital signs showed a temperature of 38°C, heart rate 168 beats/min, respiratory rate 52 breaths/min, and oxygen saturation 97% on room air. Capillary refill time was approximately 3 seconds, with mild peripheral mottling and decreased activity. Mild bilateral lower-limb edema was also noted, likely related to hypoalbuminemia secondary to systemic inflammation. Serum albumin was 2.8 g/dL. The edema resolved during clinical recovery. Abdominal examination revealed no tenderness, distension, or palpable masses.

Laboratory evaluation revealed leukocytosis (WBC 22400/μL) with neutrophil predominance and an elevated C-reactive protein (CRP) level of 45 mg/L (reference value in infants typically < 10 mg/L), consistent with an inflammatory process. D-dimer was elevated at 1050 ng/mL (reference < 500 ng/mL). However, platelet count, prothrombin time, and fibrinogen levels remained within normal limits, with no evidence of disseminated intravascular coagulation.

Abdominal ultrasonography revealed diffuse colonic wall thickening, with associated small bowel dilatation and reduced peristalsis. (Figure 2) Routine stool cultures were obtained and were negative for common bacterial pathogens including *Salmonella*, *Shigella*, and *Campylobacter*. Repeated stool microscopy was negative for ova and parasites. Stool PCR testing detected *C. difficile* toxin genes tcdA and tcdB. Histopathological examination confirmed a complete colonic mucosal cast, consistent with severe colitis (Figure 3).

Empiric antimicrobial therapy was initiated on admission with meropenem (20 mg/kg every 8 hours) and gentamicin (5 mg/kg once daily) due to concern for severe bacterial infection, in accordance with neonatal sepsis protocols. Blood cultures were obtained before antibiotic administration. Following confirmation of *C. difficile* infection, intravenous metronidazole (10 mg/kg every 8 hours) was administered, and the empirical antibiotics were discontinued. Oral vancomycin was not feasible due to the patient's inability to tolerate enteral therapy.

Clinical improvement was observed within 72 hours of initiating targeted therapy. Fever resolved, stool frequency gradually decreased, and inflammatory markers showed progressive improvement. By day 5, the infant demonstrated improved feeding tolerance and declining leukocytosis. The infant was discharged after 10 days in good condition.

## Discussion

Expelling rope-like rectal material is exceptionally rare in infants. Because infants have immature immune responses, fever and diarrhea may be nonspecific, which can complicate early diagnosis of gastrointestinal disease. The infant's severe condition, alongside clinical, lab, and radiological data, required systematically assessing and excluding multiple common causes of severe colitis based on the patient's unique presentation and progression.

Intestinal parasitosis was initially considered because of the worm-like appearance of the expelled material. However, repeated stool examinations using standard microscopic techniques revealed no ova, larvae, or parasitic elements, allowing this diagnosis to be definitively ruled out [5].

Intussusception, a frequent cause of acute abdomen in infants, was another major consideration. Although abdominal ultrasonography demonstrated colonic wall edema, the pathognomonic "target sign" was absent. Moreover, classical clinical features such as currant jelly stools and a palpable abdominal mass were lacking, and the presence of a marked systemic inflammatory response further argued against a purely mechanical obstruction [6].

Allergic colitis was also evaluated given the patient's age. This condition typically presents with mucus or blood in the stool and frequently responds to dietary modification. In contrast, our patient showed rapid clinical improvement following antimicrobial therapy, not dietary changes, and successfully resumed the original feeding regimen, making this diagnosis unlikely [7].

Necrotizing enterocolitis (NEC) was excluded based on both clinical and radiological criteria. The infant did not meet Bell's staging criteria and lacked hallmark findings such as apnea, pneumatosis intestinalis, or portal venous gas. Additionally, the patient's age was atypical, as NEC predominantly affects premature neonates [8].

Very early-onset inflammatory bowel disease (VEO-IBD) was also considered in the differential diagnosis. However, the acute clinical presentation, histopathological findings consistent with pseudomembranous colitis, and the rapid clinical improvement following antimicrobial therapy strongly supported an infectious etiology rather than a chronic inflammatory disorder [9].

After exclusion of these major differential diagnoses, pseudomembranous colitis (PMC) emerged as the most plausible diagnosis. According to the diagnostic framework described by Salen et al. [2], PMC is diagnosed based on a combination of compatible clinical features (most commonly antibiotic-associated diarrhea and fever), microbiological evidence of *C. difficile* toxins, and characteristic endoscopic or histopathological findings. In our case, the infant's critical condition and the risks associated with invasive procedures precluded colonoscopy, biopsy, and extensive serological testing [10].

Toxin enzyme immunoassays have limited sensitivity compared with nucleic acid amplification tests. Therefore, negative toxin assays do not exclude infection when clinical suspicion remains high [11]. Nevertheless, multiple lines of evidence strongly supported the diagnosis of PMC. Radiologic findings demonstrated colonic wall thickening, laboratory studies indicated a severe inflammatory response, and histopathological examination of the expelled material revealed complete mucosal detachment and sloughing. This mucosal cast, which was initially mistaken for a parasitic organism, represented a striking manifestation of pan-colonic involvement typical of severe PMC.

Despite the inability to perform lower gastrointestinal endoscopy, a polymerase chain reaction (PCR) assay for the *C. difficile* toxin genes tcdA and tcdB was performed and returned positive, thereby confirming the presence of toxigenic *C. difficile.*

It is essential to note that in early infancy, PCR positivity for *C. difficile* may indicate colonization rather than true infection. In this case, however, the diagnosis was supported by the overall clinical, radiological, and histopathological findings rather than PCR results alone. This timely diagnostic pathway was instrumental in guiding appropriate treatment and ultimately proved life-saving for the patient.

Given the infant's critical condition and initial presentation suggestive of severe systemic infection, empiric broad-spectrum antimicrobial therapy with meropenem and gentamicin was initiated according to neonatal sepsis protocols while awaiting culture results. Due to the patient being kept nil per os (NPO), oral vancomycin—recommended for severe PMC—was not feasible and was therefore replaced with intravenous metronidazole, consistent with current treatment guidelines for such circumstances [12].

The remarkable resemblance of the expelled mucosal cast to a parasitic structure underscores the importance of maintaining a strict evidence-based approach in clinical practice. Misinterpretation based on visual appearance alone risks diagnostic error and may lead to inappropriate management.

This case highlights several critical lessons. First, expelled rope-like material should not be assumed to represent a parasitic infection without definitive microbiological or histopathological evidence. Second, misdiagnosis in such critically ill infants can be catastrophic and may delay life-saving therapy.

To our knowledge, reports describing expulsion of a colonic mucosal cast in association with pseudomembranous colitis in early infancy remain extremely rare.
